# Soft Skills Are Hard Skills—A Historical Perspective

**DOI:** 10.3390/medicina58081044

**Published:** 2022-08-03

**Authors:** Silvia Iorio, Marco Cilione, Mariano Martini, Marco Tofani, Valentina Gazzaniga

**Affiliations:** 1 Unit of the History of Medicine, Department of Medical-Surgical Sciences and Biotechnologies, University of Rome, 00185 Sapienza, Italy; valentina.gazzaniga@uniroma1.it; 2 Department of Biomedical Sciences, University of Modena and Reggio Emilia, 41100 Modena, Italy; marco.cilione@unimo.it; 3 Department of Health Science, University of Genoa, 16100 Genoa, Italy; mariano.martini@unige.it; 4 Department of Human Neurosciences, University of Rome, 00185 Sapienza, Italy; marco.tofani@uniroma1.it

**Keywords:** soft skills, deontological tradition, history of patient–physician relationship

## Abstract

The increasingly swift changes in the field of medicine require a reassessment of the skills necessary for the training of technically qualified doctors. Today’s physicians also need to be capable of managing the complex issue of personal relationships with patients. Recent pedagogical debates have focused on so-called “soft skills”, whose acquisition is presented in literature as a quite recent addition to medical studies. Moreover, the historical investigation of deontological texts dating from the mid-nineteenth century back to the Hippocratic Oath shows that medicine has always discussed the need to integrate technical expertise in medicine with specific personal and relationship-based skills. Debates have often circled around whether these “soft skills” could actually be taught or how they could be successfully transmitted to training physicians. The belief that defining medicine is more complex than defining other similar sciences and that the instruments to be used in the relationship with patients cannot be limited to those provided by technical aspects shows a new awareness. Today, this view is often stated as an innovative realization on the part of doctors with regard to the complexity of training and action in a delicate area in which they are entrusted with the management of the balance of the system that is the human body.

## 1. Introduction

Unlike other sciences, medicine has always been faced with a complex epistemological order and structure. Since its early foundations as a rational discipline in Hippocratic thought, this aspect has stimulated the development and structuring of an internal thought process on the possible way to reconcile its electively scientific nature (from the Galenic definition of medicine as *logos* to the experimental method fully adopted by mid-19th century medicine), with the “humanistic” dimension of caring for patients. The latter imposes a constant confrontation with the subjective characteristic of the experience of illness and the necessary balance between the “truth of the doctor” on the one hand and the “truth of the patient” on the other [1].

Can medicine be seen as a *téchne*, a science or a “human science”? Reaching a single clear-cut answer to this question is far from easy, especially in the long term. An internal discussion on the uncertainty of medicine gains strength if we begin with a series of works dating back to the early Modern Era. This literature shows the need to mediate between the theoretical aspects of ancient traditions and the empirical approach. Even back in the 1800s, there was the complete awareness of an uncertain constitutive epistemological make-up, based on probability and the individual variability of patients. Within this mindset, we should not see disease in the abstract sense and with generalizing terms. As the famous Bolognese clinician Augusto Murri recalls, even nosology—providing important tools in order to catalogue the construction of diagnostic thinking—runs the risk of constructing abstract categories. This would lead us to neglect all those “non-technical” and specific aspects of each individual clinical situation that instead allow for the true understanding of the disease as a continuous process and change within the patient.

*Try not to get used to thinking abstractly about the disease as the pathology describes it … The more you know, the more you can* [do]. *But do not even dream to imagine that knowledge is enough. You have to get used to considering the chapter dedicated to a certain disease by pathologists neither more nor less than a necessary expedient to better understand, but not as a description of a defined and always equal entity to itself… as there are not two equal things, so there are no equal patients* […] *Thereby, even after you have made the diagnosis of typhoid fever or any other illness, you have to make the true special* [specific] *diagnosis in your patient, day by day*.

[2]

In his renowned study, “The Birth of the Clinic”, Michel Foucault [3] critically recognizes how the nucleus of medicine is based on a *logos* of empirical “visibility” of the body and disease. Scientific medicine bases its view on the empiricist paradigm that allows us to look at the body as a thing in a world of things. Clinical method is based on visual examination. Signs and symptoms are related to the repetition of the frequency of illness. Therefore, decrypted from the individual meaning, these signs and symptoms become meaningful. Consequently, pathology assumes a pre-defined meaning.

*It is the description* […] *that authorizes the transformation from the symptom into sign, the passage from the sick person to the disease, access from the individual to the conceptual.* […] *Describing* [this] *means following the order of its manifestations, but it also means following the intelligible sequence of their genesis. It means seeing and knowing at the same time, because, by saying what one sees, one spontaneously integrates it into knowledge. This also means learning how to see, because this means giving the key to a language that masters the visible*.

[3]

Along these lines, disease is bound to the perception of a mechanistic visibility. Therefore, it is entirely disconnected from the world of invisibility that builds disease and comes together to create it. An entirely empiricist view ignores the fact that human beings are not just bodies but symbolic and social animals, whose existence is understandable in its multiform manifestations. Disease is obviously part of this. We can reach this multifaceted understanding only if we consider the social and cultural determinants that contribute to shaping all individuals of our species in extremely different ways.

Furthermore, leading contemporary definitions, such as that of G. Canguilhem in 1988 [4], underline how the nature of medicine oscillates between the clinical world, aimed at taking care of the individual or a part of the population, and the need to increase our level of scientific understanding by making use of basic knowledge and methodologies addressed at the mere explanation of general pathological and clinical aspects.

A solution to this ontological dilemma appears to be at least partially offered by medical anthropology. Furthermore, this field of study offers a possible categorization of medicine as a form of human science and specialized culture. Based on behaviour that is taught and communicated, medicine has all the characteristics that define subcultures. Medicine has a peculiar symbolic system of verbal and non-verbal communication, direct and indirect self-imposed limits, as well as shared ideal models that create behavioural codes. Moreover, medicine has evolved over time in an adaptive way in order to best apply this expertise to the needs of a certain context. Consequently, it is integrated within the cultural context of reference of which it exploits and influences different aspects of life, changing due to innovation and direct and indirect transmission. Finally, medicine selectively spreads and divulges intuitions and discoveries through assimilation, acculturation and revolutions. Each of these steps is so closely interconnected with what happens in specific societies that it is difficult, if not impossible, to understand the profound changes of medical thought outside the cultural contexts in which it is generated.

## 2. Soft Skills in Medicine?

The rapid transformations of social and working contexts increasingly require medical education to design innovative paths that include, alongside the acquisition of fundamental technical skills (hard skills), also the construction of transversal skills, which today are often coined as “soft skills” [5]. The medical–pedagogical debate has been recently enriched by important contributions, in part aimed at identifying an agreed definition and a shared denomination for soft skills in medicine and in at defining the minimum “humanistic” skills that are needed during the training and studies. This recent discussion is also aimed at focusing on the goals that teachers and trainers aim to obtain through the planning of an innovative syllabus that is also directed towards the acquisition of these “non-technical” skills.

Often called generic skills, essential skills, key competences, employability skills, emotional quotient skills and life skills, these terms have been grouped within all those abilities for adaptive and positive behaviour, which enable individuals to deal effectively with the demands and challenges of everyday life [6]—traits and capabilities that do not substitute but rather substantially integrate a professional skillset, allowing the future doctor to develop essential expertise for personal development, social participation and workplace success. Moreover, these skills ensure that doctors have these “intangible” abilities that are essential for strengthening their professional profile as communicators, leaders and negotiators [7].

Literature has recognized the challenge of identifying a thorough definition of soft skills in medicine and their vast extension over different fields and cultural areas. They develop on complementary levels, ranging from communication and interpersonal skills to their use paired with the development of generic constructs—i.e., emotional skills as in the classic definition by D. Goleman [8] eventually leading to self-motivation skills, leadership and organizational skills. These aspects must also be combined with those of the development of scientific literacy, critical thinking and problem solving, which fall at least partially in the domain of the acquisition of emotional competence that favours communicative exchanges in emotion-eliciting social transactions. In a nutshell, we are dealing with a basic understanding of the ethics of the doctor–patient relationship—the ability to face diversity, articulated on the different levels of anthropological thinking and an epidemiological approach to health based on the themes of fairness in health and healthcare. From medical pedagogy to behavioural psychology, as well as work psychology, bioethics and medical anthropology, many cultural contexts are involved in order to allow for the development of skills that enable the future doctor to “navigate in the sea of complexity” in which we find the clinical approach and the management of the doctor–patient relationship. The adoption of soft skills would allow for the training of resilient physicians, able to sustain stress and manage in a creative and simultaneous way the changing reality of stories of illness told by different patients in varying contexts. This includes their psychological situations, the relationships with their team and their ability to deal with a social context in which requests and expectations are exponentially increasing [7].

Among the advantages that the literature indicates are expected from the acquisition of soft skills, we find the dualism of the personal advantage of the professional (attribution of sense to daily work, management of the routine aspects of the medical professions, contribution to overcoming logistic difficulties work organization) and the improvement of patient satisfaction. In medicine today, complaints rarely concern an incorrect or lacking application of technical knowledge or the lack of professional skills. Instead, patients are increasingly focused on the lack of communication, the emotional barrier and their perception of the doctor as a distant figure who is often unwilling to create an emotionally satisfying dialogue [9]. Consequently, soft skills are a fundamental not only as a tool for reducing the risk of burnouts but also as a tool in the hands of doctors to reduce legal disputes and accusations of incompetence, imprudence or malpractice.

Soft skills are difficult to actually list, quite difficult to learn and even more difficult to assess. Their learning appears to take place, for the most part, in line with a cultural model of learning by assimilation or imitation. The concept of hidden curriculum, valid both in a positive and “non-positive” sense, presupposes a central role of “indirect” pedagogy. In this sense, virtues based on personality and behaviour, openness towards the patient, the physician’s ability to manage stressful situations and the difficulties of interdisciplinary and teamwork, come together to become, on a case-by-case basis, models to be introjected and taken in or rejected in some situations.

## 3. We Need Soft Skills in Medicine—Is This Really a Contemporary Debate?

The concept of the therapeutic alliance between doctor and patient in the fight against disease goes back thousands of years. The image of the crater of Sosias (5th century BCE) shows an interesting inversion of roles in which Achilles becomes Patroclus’ *therapon* in his care. Moreover, the word *therapeia* is connected to the concept of service in its etymology, and the word’s meaning is more an act of caring for someone, rather than the specific act of curing someone. In this sense, the paternalism traditionally attributed to the doctor undergoing Hippocratic training [10] seems to lose consistency when it comes face to face with the actual role of *therapon*, which the doctor takes on in the relationship with the patient. Furthermore, the concept of *therapeia* implies a predisposition to empathy and to obedience in the sense of *ob-audire* (listening). This is essentially a virtuous combination of listening and supporting, aimed at a restitutio ad integrum that is compatible with the patient’s lifestyle and expectations [11]. The centrality of the patient in the act of care, reported in the texts of the *Corpus Hippocraticum* as a crucial moment of a relationship in which the doctor has the sole function of supporting the fight against the disease that weighs primarily on the patient, returns in history of Western medical thought with an apparently singular alternation of attitudes. From time to time, the patient-centred approach becomes a tool of strength to interpret the signs of the disease and reconstruct them within a logically coherent conceptual framework (as seen in Hippocratic thinking) or an element of weakness, capable of affecting the “high status” of art by introducing an element of misleading and harmful subjectivity for the purpose of constructing clinical discourse (as we can see in part of Galenic thought and in many texts of the subsequent medical tradition inspired by this). In short, the so-called Hippocratic triangle [12] (whose vertices are constituted by the constant relationship between doctor, patient and illness) constitutes the first moment of an explicit thought process on the role and importance that the subjectivity of the illness experience can have in the image that medicine aims to transmit to society. Moreover, the same Hippocratic definition of medicine as *téchne*, meaning the ability to produce a tangible good (health in this case) by understanding the ways in which the body functions and things happen is the first systematic attempt to find a satisfactory response to questions about the true nature of a field that does not end simply within the mere definition of a science.

The belief that defining medicine is more complex than defining other similar sciences and that the instruments to be used in the relationship with patients cannot be limited to those provided by technical aspects shows a new awareness. Today, this view is often stated as an innovative realization on the part of doctors with regard to the complexity of training and action in a delicate area in which they are entrusted with the management of the balance of the system that is the human body.

In the eyes of the historian, this awareness is actually not so innovative. Let us try to offer a short explanation that retraces some salient stages of medical thinking—which we could define in a generic deontological way, even if its message certainly does not end in the sphere of behavioural legislation—in which we find the constant reference to the dualism of the need for training that is technical and “human” at the same time. We will start from texts dating back to the 18th and 19th centuries and then head back to the past, with a rather brief discussion on periods that are quite unique, up until the birth of rational Hippocratic medicine.

An interesting starting point can be found in the reading of the *Galatei medici*, small manuals on medical etiquette that were published throughout Europe at the turn of the 18th century and the middle of the following century. Conceived in order to enhance the figure of the physician in an age in which other health workers were laboriously attempting to climb the academic ladder (e.g., the first constitutions of obstetric and nursing groups), the *Galatei* manuals represent above all a tool to spur the doctor to endow themselves with the self-awareness necessary for the social affirmation and the gain of public confidence. The texts of the *Galatei* repeatedly state that the choice of the doctor should be made on the basis of good moral and personal skills or knowledge and expertise that he possesses and that only the recourse to the dualism of science and humanity can guarantee the acquisition of the trust and confidence necessary for the establishment of a correct doctor–patient relationship. The prologue of the *Galateo* for G. Pasta, published in Rome in 1817, also highlights laws known to the medical community. These laws were learned through science, but they are also “dear” to patients by virtue of human and behavioural qualities that form the basis of a code of civilization, which can also be seen as medical “etiquette”. Science without etiquette (where the word indicates the highest and morally connoted deontological world) makes us glorious but only for half of it [13]. Moreover, etiquette requires a moral character on which to be grafted, a personal nature that is “prosperous, grave, eloquent, tireless, docile, civil”, which makes the doctor capable of acting prudently, relating with colleagues with cordiality and interest, and in the relationship with the patient, turning into a friend who is also capable of encouraging and comforting. The “decorum” proposed by the *Galatei* texts is not confined to appearance, which must also be impeccable. There is a substance, a fidelity to the generous and human aspect of the doctor’s soul, which must be nourished by non-technical instruments, but which are equally fundamental. Among these, the knowledge of languages, poetry as a tool for setting and controlling the quality of language, the possession of logical tools that allow us to “observe a great deal, but rightly observe”, the knowledge of the world, as well as character traits that condition the patient’s response to conditions of illness, paired with tolerance to diversity and the ability to apply correct behaviour to different social conditions directly recall a modern definition of soft skills.

Reference to the need for a complex and thorough medical education is actually a quite classic theme. It constitutes the pivot of a well-represented pedagogical tradition, in modern times, from the famous text *Dissertatio de recta medicorum studiorum ratione instituenda*, work of the pontifical archiater Giovanni Maria Lancisi, printed in Rome in 1715 [14]. Lancisi’s work is a tried-and-true programme for the training of doctors and surgeons who work inside S. Spirito, the Roman hospital at the centre of an innovative cultural project, commissioned by Pope Clement XI and entrusted precisely to the realization of his archiater. The project foresaw to the establishment of a hospital library, which would gather the most significant and most recent medical, scientific and philosophical texts, in order to make them available to those who worked inside the hospital and needed a “continuing education”. This led to the establishment of an academy, the centre of the hospital’s “experimental” life and a forum for debate and scientific meeting. Moreover, the hospital drafted a complete educational project for the doctor in training, expressed in the *De recta*, which was based on the extraordinary possibility offered by the hospital to create a channel of communication among different professionals, allowing them to meet many patients simultaneously and to consult a vast collection of books. Lancisi firmly believed that the doctor, in addition to being endowed with all the most innovative cultural instruments of the time (the knowledge of mathematics, mechanics, chemistry, etc.), must also be trained in the acquisition and exercise of specific relational skills. They must know how to speak, not because eloquence is able to cure but because it is an indispensable tool to convince the patient and engage their trust, in the same way as the dignity of manners and the authority of behaviour are. Furthermore, they must know the uses and customs of the world, nourish charity as the main instrument of approach to the disease—a precursor that religiously connoted our concept of empathy—in order to exercise with prudence and reason what should be done. The concept was that if something is completed quickly, it can help avoid other problems. A physician must also apply a clear-cut logical method, which allows them to illuminate the “dark ravines” of the reflection on the nature of the body. The construction of this method requires some tools, not all provided by technical studies. It feeds on knowledge of the world acquired through travelling, experience and mental journeys in books and illustrations (the paper world, for those who—like Lancisi—founded a library, is equivalent to the geographical world). Clearly, the physician needs doctrine, but they should also have a good and generous nature, willing to be shaped, from childhood, by education, working with dedication and “continuous time”. For Lancisi, the possession and cultivation of apparently secondary abilities is not to be understood as a decorative tool. These skills are an indispensable qualification, due to the fact that medicine, which “*scientiam sibi vero comparare volet*” (which aspires to become a science, which aims to be compared to a science) can lay the groundwork to legitimize the aspiration to implement its epistemological status. That said, some small mistakes must be avoided. Certain cultural trends of the time (*polymathia*, wanting to know a little about everything, for example) should be avoided, just as the excessive use of artistic and cultural activities (poetry, theatre, music, antiques). This trend would end up becoming a distraction for training doctors, who must not lose their clarity, perhaps obscuring good reasoning with an excess of rough and confusing notions. However, none of the stimuli that come to us from these examples of early modern and late modern medical literature is entirely new to medicine. Some of these features are defining aspects in older works, ascribable to Greek and Latin medical literature, which paved the road to Western medicine starting in the late ancient period. Galen’s beliefs on the need for the doctor to also be a philosopher, holding a solid knowledge of logic, physics and ethics, led to the development of an ontological foundation for medicine in the Imperial Age, anchored to the dualism of science and philosophy. In a mutual process, medicine and philosophy lend to each other their own methodological tools, so that without scientific basis even philosophy is reduced to a mere rhetorical exercise and a series of unmotivated controversies [15]. Pedagogy, to which Galen devotes a large part of his writings, is depicted in the same treatise as a maieutic process in which the master guides the pupil. The student’s views are rendered acute by experience and acquired knowledge, towards the contemplation of intelligible objects. In medicine, this translates into the acquisition of the experience data necessary to formulate the prognosis, through their selection and re-composition within a coherent logical framework that explains the onset of the disease [16]. The student’s conduct towards the acquisition of skills is not only mediated by the teacher’s more developed insight but also by a series of conditions that fall entirely within *our* definition of soft skills: an early and constant exercise in fundamental skills; possessing a “penetrating nature” and a naturally curious disposition towards the leading intellectuals of the time, and the constancy of applying oneself to continuous study; absolute dedication to work; the intellectual tension that leads to the search for truth through the possession of a method that allows to discern what is true from what is false; and lastly, daily exercise in this method, so as to master it and know how to apply it with satisfactory results [17].

The Galenic attitude towards the patient, often quite stern and connected to an absolute and rigid paternalism, does not indulge in defining the doctor in terms of human qualities. However, this attitude is compensated, in ancient literature, by an entire series of concepts summarized in the famous figure of the *medicus amicus*. Medical sources (Celsus and Scribonius Largus, 1st century CE) and literary sources (Seneca and Cicero) repeatedly describe the human qualities necessary to be a good doctor. Celsus expressly states that, with the same skills, it is preferable to have a doctor who presents himself as a friend rather than as a detached and extraneous professional. Furthermore, he adds that mercy, which is the attitude of the soul that opens up to the patient’s understanding, is a tool that can improve the performances of medicine [18]. Scribonius Largus, for his part, emphasizes that a fundamental cornerstone of medicine is the foundation of the will with which medicine moves forward [19]. Consequently, this will cannot be excluded. At times, the tradition goes a step further. *Quaestiones medicae*, attributed to Soranus, puts forward an opinion ascribed to the Alexandrian doctor Erasistratus, which affirms that, despite the fact that the combination of professional expertise (in arte perfectus) and human virtues ](moribus optimus) certainly represents the ideal condition when imagining the perfect doctor, in the absence of one of them, “it is better to be a good man who lacks in doctrine (absque doctrina) rather than being a perfect technician with a bad personality, good virtue” [20].

These technical voices are echoed in the passionate words of famous patients such as Seneca and Cicero. Their testimony gives life to those “using” medicine. These voices are often evanescent, mute and difficult to reconstruct in all Western medical literature. For them, those who lose a doctor’s human capacities are left orphans of a skill that money cannot buy and which cannot be replaced even through the use of a more perfected technique. What the patient asks is time dedicated to him by the doctor, choices of his case compared to the many others who request his presence, promptness of intervention and solicitude in assistance, as well as the ability to bear the demands of those who suffer, who can often be bothersome. All this constitutes the real treasure that medical art can offer—a human skill that makes the professional irreplaceable. The perfect doctor is one who knows that the occurrence in the same body of the patient and his or her illness do not constitute an identity as such [21].

## 4. Conclusions

The need for the patient to have a physician who has studied but at the same time is highly qualified in human relationships boomerangs back in literature and testimony from contexts that are sometimes unexpected, such as social and administrative situations. For example, Greek epigraphic tradition continually attests that the criterion of choice of the public doctor suitable to take care of the city must be defined based on the evaluation of technical excellence as well as an appropriate moral attitude. A decree of the sanctuary of Asclepius in Athens (dating between 46 and 125 CE)—or the numerous decrees that were dedicated throughout the territory of Greece to doctors known to us only for the brief mention of their name—highlight the medical *episteme* to the *ethos*, that is the set of lifestyles, customs and moral attitudes that make professionals appreciated candidates for the management of public health.

The relationship between professional skills and an individual’s personality traits has been clearly attested since the Hellenistic tradition. Moreover, on a consistent basis, this relationship underlines the main features of the Hippocratic *ethos*: the love of mankind, the foundation of medical art, constitutes one of the main tools thanks to which the sick regain health. The theme emerges with a certain clarity in the second corpusculum of the Hippocratic treatises dedicated to medical ethics: *Medico, Precetti e Decoro* (III century BCE - II century CE) [22]. The aspect that unites these writings is the identification of the good doctor in a balanced pairing of philanthropy and authority. However, they also offer practical indications on the appearance and demeanour that doctors are called upon to carry when they are in front of the patient. This is important not only to avoid discrediting themselves and their profession but also in order to instil a feeling of trust in the patient [23]. Moreover, returning to the text of the oath ascribed to Hippocrates, the combination “life/art” already appears clearly defined in the statement “I will preserve my life and my art”. The problem of the interpretation of the Greek term “*bios*”, so connoted in the text of the oath, can be solved only if, with von Staden, we agree to translate it as the way of life, the styles and the totality of the actions that shape a human being as such—a doctor as a doctor, a *good* doctor as a *good* doctor. Therefore, we are discussing not only science but also the set of personality trains and acquired attitudes that today fall within the domain of soft skills. In conclusion, due also to the therapeutic weakness of medicine, in the historical tradition, these “human” traits were never described as some sort of accessory [24]; rather, they are fundamental elements in creating the role of the suitable professional. Perhaps our thoughts could also wander to “*Homo bonus*”, created by the hands of an anonymous patient somewhere around the III century BCE. This graffiti is a sort of thank you note written on the wall of the visiting cubiculum of the surgeon’s domus in Rimini, Italy. This is a representation of that luminous figure that passes through the centuries in order to help the “sufferers” who invoke his help to win their battle against illness.
